# Ethics

**Published:** 1896-05

**Authors:** S. B. Brown

**Affiliations:** Ft. Wayne, Ind.


### THE DENTAL REGISTER.

VOL. L.] MAY, 1896. [No. 5.

### COMMUNICATIONS.

### Ethics.

BY S. B. BROWN, D D S., FT. WAYNE, IND.

*To the Students of the American Dental Colleges:*

It is an esteemed privilege to offer congratulations to the dental students of America upon their wisdom in electing to become dentists.

No other department of human endeavor offers greater fields for usefulness or greater reward to the faithful.

It is a source of gratification with our profession at large to note the advanced methods of teaching, and the higher intellectual standard of our pupils to receive it.

The increasing number of dental colleges, with their multiplicity of graduates, make evident the fact of increasing demand.

If this be true under existing conditions, where a surprisingly small percent of our population seek the services of a dentist save for the extraction of teeth, what would be the demand if all students could be effectually educated and conscientiously impressed with their sacred duty to their profession and the world, which eschews the extraction of teeth and uncompromisingly strives to preserve them?

A paramount question which should be discussed before the humblest and highest dental society is, "Why do Dentists Extract Teeth?"

Why do those who have faithfully passed through the curriculum of a dental college, and perchance made sacrifices of time and money, oftimes drawing upon the resources of family and friends to acquire a full knowledge of the diseases which human teeth are heir to, and treatment for their restoration, for a careful study of the Divine Architect's infinite wisdom in their development for the physical perfection of creatures made in His own image? Why do they stultify the good name of dentistry by an assault to annihilate these priceless organs with forceps, and at the same time casting away that dearly acquired education for their presrvation and any reputation which their profession is presumed to have as conservators?

Can any one dispute the importance and profit of a serious consideration of this question?

Does it occur to some of my listeners to ask, Is this subject pertinent to ethics?

The reply to such a suggestion is, that from long observation and study of the subject, extracting teeth, as practiced by many dentists, is an evil which is synonymous with devil, and is a veritable satan stalking abroad in our profession seeking whom he may beguile.

This temper has perverted a normal sense of justice and honor and so dwarfed self-respect, when yielded to, that nefarious methods are adopted for supposed gains.

These methods are the sandy foundations upon which to build the practice of the "advertising dentist." Printer's ink, gas bag and hypodermic needle are his chief material accessories, with these he decoys the ignorant, innocent and unwary to his selfish, conscienceless course.

Whatever else his "dental dodgers" may claim for him in skill is secondary to "cheap, painless extracting."

By these unethical and misleading methods the advertiser invades a community, and aims to prejudice public opinion against regularly educated, reputable and established dentists as extortioners and incompetents, and by his guerrilla methods is willing to traffic in the good name of a profession from whose store-house of lore he has drawn all that gives him a name which he now dishonors by his treachery.

He is willing to influence citizens to the belief that other dentists who are united and mindful of professional courtesy are a combination for the purpose of extorting unjustifiable fees—that dentistry is a trade and not a profession—that dental skill is over-paid and does not deserve the reward attending conscientious labor for years to establish, and to which he would be cordially invited to share if he would add his mite to maintain that confidence and respect due an honorable profession in which he claims a brotherhood, and more to render a duty to humanity by a higher standard of skill in demonstrating professional truths—to educate and encourage the people to seek for their benefit the grand possibilities of modern dentistry.

In this light who will say his methods are not false?

The fallacious defense of the quack is that the established and regularly accepted plan for securing a practice is too slow, that he is too enterprising to wait, or his pecuniary necessities forbid it.

Thus he is willing to lower the standard of benefits to all others for his supposed individual gain, to subordinate every other high purpose to his selfish end.

Are not ethical questions involved in this course?

The next vain effort to vindicate a weak and wicked course is that the people will not accept treatment necessary for the conservation of diseased teeth, that they demand unconditional extraction; if one does not comply another will, therefore, it is justifiable to secure the profit of this ill-advised procedure.

"The people demand it." Do we admit that we should take our professional orders from those who are totally ignorant of the first principles in what many years have been spent by us in acquiring to become competent authority in regard to the proper treatment? Is it to count as nothing? Is this diploma won only for a license to conduct a dental slaughterhouse?

Shall our patients dominate our course of treatment against our knowledge of right?

Oh! dentistry, what sins are committed in thy name.

Webster defines ethics as the science of human duty, a duty incumbent upon all to respect the rights of others in their daily intercourse, to subordinate individual interests to the common good. This is essential to all advance in civilization and social evolution, and doubly true in advancing proficiency in our profession.

It is assumed in the professions that individual learning and social culture has its highest representatives, and when one falls below the ethical standard his degradation is strikingly apparent.

From our vantage ground moral impulses to just acts should take us far beyond the need of admonition of a written code of ethics as it does beyond the need of statutes against larceny and malicious trespass, these offenses having no parallel with the broken spirit of the unwritten code of ethics. I quote them for your comparison.

### GRAND LARCENY.

Whoever feloniously steals, takes and carries, leads or drives away the personal goods of another, of the value of twenty-five dollars or upward, is guilty of grand larceny, and, upon conviction thereof, shall be imprisoned in the State prison not more than fourteen years nor less than one year, fined not exceeding double the value of the goods stolen, and disfranchised and rendered incapable of holding any office of trust or profit for any determined period.

### PETIT LARCENY.

Whoever shall feloniously steal, take and carry, lead or drive away the personal goods of another of the value of any sum less than twenty-five dollars is guilty of petit larceny, and upon conviction thereof shall be imprisoned in the State prison not more than three years nor less than one year, fined in any sum not exceeding five hundred dollars, and disfranchised and rendered incapable of holding any office of trust or profit for any determined period, or he may be imprisoned in the county jail not more than one year, and be fined in any sum not exceeding five hundred dollars and disfranchised and rendered incapable of holding any office of trust or profit for any determined period.

Upon a second conviction of petit larceny, the person convicted shall suffer the punishment prescribed for those convicted of grand larceny.

MALICIOUS TRESPASS.

Whoever maliciously or mischievously injures or causes to be injured any property of another or any public property is guilty of malicious trespass, and upon conviction thereof shall be fined not more than twofold value of the damage done, to which may be added imprisonment in the county jail for not more than twelve months.

The question is submitted, Is not the invasion of the advertising dentist into any community a greater offense than the committal of crimes here quoted, and made punishable by fines and imprisonment? The larceny of twenty-five dollars has no part in comparison with the wrong perpetrated by the class here held in view.

One who has become sufficiently degraded to commit the least of these offenses whereby imprisonment is a possible penalty would be marked as unworthy of confidence in moral rectitude, likewise he who subordinates moral principle to base avaricious desires, as in the methods of the "advertising dentist," so weakens his better perceptions that moral turpitude stain his life —a fallen man, fallen below the ethical law of plain human duty. Where ethical law ends, common law begins. Is he who ignores the former safe in standing just beyond the grasp of the law for recognized wrongdoers—only on the borderland of outlawry?

Benjamin Kidd declares that "one who brushes aside the restraints of moral principle is little more than a highwayman at heart."

Individual interests can not progress to their best development without subordination to the best interest of humanity. "As well might we argue that because the fruit survives for a time when removed from the tree, and even mellows and ripens, that it was therefore independent of the tree." "Only by generating that great fund of altruistic feeling can all people be ultimately brought into the rivalry of life on conditions of equality." Nothing short of a weakened moral perception can be attributed to those who parade their claims for superiority and cheap operations in the advertising press. A newspaper advertisement of more than your name and location is superfluous.

Your sign and a standing professional card is all that can be of substantial aid. Intelligent persons seeking the services of a dentist are more diligent than you may suppose in gaining information of your character, and are not influenced in your favor by paid-for and self-dictated compliments.

In your office alone can you make the reputation which is desirable and permanent. Only here, by your good works, can you spread your fame through well-pleased patients, who will be glad to advertise and recommend you from their hearts.

Fees commensurate with the time and skill involved in your labor must be demanded. To ask less is sure to lower your standard of excellence. Such has been the progress in the science of human duty and endeavor that a new era is rapidly dawning upon us. We are rapidly rising above the requirements of self-imposed restraints in a written law of ethics, as we are above the State criminal code. The unwritten law of ethics, the limits of which include the Golden Rule, is our only guide and security. When we recognize this we will be as far above any personal concern for the written code of ethics as we now are above the State criminal law, and will regard violation equally repugnant.

All who are ordinarily observant must recognize the fact that we are now living in what may be termed an awakening era. A greater force of thought as to how we can better live is coming to us. The best and purest minds of this age are making a searching analysis of the great questions of life—of time and eternity, mortality and immortality. These scientists concur in the belief that we are nearing a pause in the material side of life—that art and invention which has made such marvelous strides in the last half of our century is now yielding, in harmony with a law of alternation, to the metaphysical or spiritual questions concerning man.

From a sense of duty I call your attention to this subject and ask that it may become a part of your inquiry and study. By so doing you will find an answer to many problems in life. You will find that earth should not be the battle-ground for brother against brother to gain subsistence. This study can alone reveal to you the real ethical law, and make it a rule of faith and practice.

The essential and supreme gifts which make up a perfect man is given by Drummond as follows: "Patience, kindness, generosity, courtesy, unselfishness, guilelessness, sincerity, humanity, good temper." When we consider that these attributes are essential to perfect manhood, how fragmentary must be our claims. Still, we must not despair, as the first named is patience.

I pass to the last, and ask you as dentists to acquire it. A good temper cannot be overestimated. It is so easy to become exasperated when everything is not as we wish it to be. In our juvenile days anger was our first demand to stimulate us to a supposed proper resentment of all aggressions. Our associates encouraged and applauded it until it grew with our growth, and after we attained to the age of better judgment our philosophy is too often unequal to its subjugation.

Anger is our besetting sin, a relic of barbarism, purely animal.

A hot temper is the expression of an unenlightened selfishness which tolerates only "my way" and has no consideration for your way. "It is never justifiable in an intelligent person; it is an original sin attended in the past with all the cruelties of wars' tortures and inquisitions, an inheritance from savagery and utterly inconsistent with civilization or intelligence."

Under its force reason yields. Rage is momentary insanity. It is a passion which intellectual cultivation should disown. Humanity is defined as kindness and benevolence. The human tongue, when unrestrained by it, is an evil greater than alcoholic intemperance, in pernicious gossip and generally disparaging remarks.

We can say in sixty seconds what many years of vain regret will fail to obliterate—inject a poison past all healing. Drummond writes that "God has stowed away a vast amount of enjoyment in the deeds we never do, and the words we never say."

The exactions of polite society impose what may be expressed as rules of propriety, and is known as etiquette. Etiquette as concerns us professionally in regard to our inititatory intercourse with our fellow practitioner is the reverse of general society's rule, in that the newcomer makes the first call upon reputable resident dentists of the place in which he elects to locate, informing them of his intention of becoming a resident and expressing his desire to be in ethical relations, when courtesies due will follow.

I rejoice at the presence of women in our ranks; they will be a potent factor in this great reformation; with their intuition of right, keen perception and delicate impulses they are certain to become barriers to all degrading deviations and lead in that refinement for which we must all aim.

In conclusion, I can sincerely give you every encouragement as prospective dentists; there is room and reward for the expansion of every noble ambition. When you enter the dental mansion, bring to the household no disorder, and by your aid we will enter upon the new century just dawning, fortified to lead the lives which infinite justice demands.

